# The complete mitochondrial genome of *Amorphophallus albus* and development of molecular markers for five *Amorphophallus* species based on mitochondrial DNA

**DOI:** 10.3389/fpls.2023.1180417

**Published:** 2023-06-21

**Authors:** Yuanyu Shan, Jingling Li, Xue Zhang, Jie Yu

**Affiliations:** Key Laboratory of Horticulture Science for Southern Mountainous Regions from Ministry of Education, College of Horticulture and Landscape Architecture, Southwest University, Chongqing, China

**Keywords:** *Amorphophallus albus*, mitochondrial genome, RNA editing, MTPT, molecular marker

## Abstract

**Introduction:**

*Amorphophallus albus* is an herbaceous, cormous, perennial plant used as a food source and traditional medicine in Asia.

**Methods:**

In this study, we assembled and annotated the complete mitochondrial genome (mitogenome) of *A. albus*. Then we analyzed the repeated elements and mitochondrial plastid sequences (MTPTs), predicted RNA editing sites in mitochondrial protein-coding genes (PCGs). Lastly, we inferred the phylogenetic relationships of *A. albus* and other angiosperms based on mitochondrial PCGs, and designed two molecular markers based on mitochondrial DNA.

**Results and discussion:**

The complete mitogenome of *A. albus* consists of 19 circular chromosomes. And the total length of *A. albus* mitogenome is 537,044 bp, with the longest chromosome measuring 56,458 bp and the shortest measuring 12,040 bp. We identified and annotated a total of 36 protein-coding genes (PCGs), 21 tRNA genes, and 3 rRNA genes in the mitogenome. Additionally, we analyzed mitochondrial plastid DNAs (MTPTs) and identified 20 MTPTs between the two organelle genomes, with a combined length of 22,421 bp, accounting for 12.76% of the plastome. Besides, we predicted a total of 676 C to U RNA editing sites on 36 protein-coding genes of high confidence using Deepred-mt. Furthermore, extensive genomic rearrangement was observed between *A. albus* and the related mitogenomes. We conducted phylogenetic analyses based on mitochondrial PCGs to determine the evolutionary relationships between *A. albus* and other angiosperms. Finally, we developed and validated two molecular markers, Ai156 and Ai976, based on two intron regions (*nad2i156* and *nad4i976*) respectively. The discrimination success rate was 100 % in validation experiments for five widely grown konjac species. Our results reveal the multi-chromosome mitogenome of *A. albus*, and the developed markers will facilitate molecular identification of this genus.

## Introduction

*Amorphophallus albus* is a perennial herb that has been cultivated in southwestern China for over 2,000 years. The underground corm of *A. albus* is considered a functional food due to its richness in amino acids and trace elements necessary for the human body. Its most notable effect is its ability to clear fat and lower sugar, making it renowned for its effectiveness in weight loss and body slimming (Devaraj et al., 2019). In fact, the underground corm of genus *Amorphophallus* can produce large amounts of konjac glucomannan (KGM). It is the only economic crop capable of synthesizing KGM, which could play a significant role in preventing and treating cardiovascular disease, delaying aging, and aiding in weight loss, among other benefits. Thus, *A. albus* has always been used as a natural health food and ideal meal for generations of people, and it is very popular in China, Japan, and other Asian regions. Despite *A. albus* having relatively smaller plants and lower yields compared with *A. konjac*, the glucomannan content in *A. albus* corms is the highest among all *Amorphophallus* species (Yin et al., 2019). Consequently, the cultivation of this species has especially great economic value. Numerous rural areas in China are growing *A. albus* as a pillar industry. It is called the silver of the plantation industry (Tang et al., 2020).

Mitochondria play a crucial role in various cellular physiological activities, serving as important sites for energy synthesis and conversion (Birky, 1995; Ye et al., 2017). Because mitochondria convert biomass energy into chemical energy through oxidative phosphorylation and are involved in cell division, differentiation, and apoptosis, they play a crucial role in plant growth and development (Kroemer and Reed, 2000; van Loo et al., 2002; Bonora et al., 2014). According to the endosymbiotic theory, mitochondria originate from endosymbiotic alpha-bacteria within archaea-derived host cells, eventually evolving into organelles of eukaryotic cells (Roger et al., 2017). In angiosperms, the nuclear genome is biparentally inherited, while chloroplasts and mitochondria are maternally inherited (Cheng et al., 2021). This genetic mechanism eliminates the influence of the paternal line and facilitates our study of the genetic mechanism (Wallace et al., 1988). As of February 2023, our query in the NCBI genome repository revealed the release of 585 mitogenomes, 10,123 chloroplast genomes, and 1,293 plastid genomes (https://www.ncbi.nlm.nih.gov/genome/browse#!/organelles/). From the data deposited in NCBI and a study of mitogenome (Bi et al., 2020), it is evident that mitogenome structure is complex and challenging to assemble. The size and structure of mitogenome in angiosperms vary greatly, with *Silene conica* having the largest mitogenome (11.3 Mb) and *Viscum scurruloideum* having the smallest mitogenome (66 kb) (Sloan et al., 2012; Skippington et al., 2015), while the mitogenomes’ overall size usually ranges from 200 to 750 kb. And some mitogenomes contain highly variable intergenic regions (Burger et al., 2003). Although most plant mitogenomes assemble into circular arrangements, the *in vivo* confirmation of plant mitogenomes is much more diverse, including branching linear structures with frequent rearrangement (Jackman et al., 2020). And some may be polycyclic, such as maize (Fauron et al., 1995) and kiwifruit (Wang et al., 2019), some may be linear, such as *Lactuca sativa* (Kozik et al., 2019). Moreover, numerous intramolecular recombination events and sub-genomic conformations have also been reported (Gualberto et al., 2014). These results indicated the instability of mitogenome structures in higher plants.

In our current study, we performed the following tasks: (1) assembly and annotation of the mitochondrial genome of *A. albus*, (2) analysis of repeated elements, (3) analysis of mitochondrial plastid sequences (MTPTs), (4) predictions of RNA editing sites in mitochondrial protein-coding genes (PCGs), (5) inference of phylogenetic relationships of *A. albus* and other angiosperms based on mitochondrial PCGs, and (6) design of two molecular markers based on mitochondrial DNA, successfully discriminating five *Amorphophallus* species. Our results provide a theoretical basis for the identification and biological study of *A. albus*, and plays a significant role in the breeding and cultivation of this species. Additionally, this work presents a novel case of multiple chromosomes for the mitogenome of plants, offering valuable insights into the evolutionary patterns of mitogenomes in higher angiosperms.

## Materials and methods

### Plant materials and sequencing

In April 2022, we visited the Xiema Konjac Germplasm Resource Park (N29°46′4.20″, E106°21′53.07″) and collected fresh leaves of five *Amorphophallus* species, namely *A. albus, A. konjac, A. krausei, A. bulbifer*, and *A. paeoniifolius*. These specimens have been deposited in the herbarium of Southwest University, Chongqing, China, with the accession number: 20220420CQ-1 to 20220420CQ-15. For *A. albus*, the total genomic DNA was extracted by using the CTAB method. The DNA library with an insert size of 350 bp, was constructed using the NEBNext® library building kit and was sequenced on the NovaSeq 6000 sequencing platform at Novogene (Beijing, China). The sequencing generated a total of 4.95 G of raw data, resulting in 16,506,686 raw reads. Clean data were obtained by using Trimmomatic (Bolger et al., 2014). We removed low-quality sequences, including sequences with a quality value of Q <= 5 that accounted for more than 50% of the total bases and sequences in which more than 10% of bases were “N”. The plant sample used for Illumina sequencing was subsequently subjected to Oxford Nanopore sequencing. Purified DNA was prepared for long‐read sequencing following the protocol in the SQK‐LSK109 genomic sequencing kit (ONT, Oxford, UK). In total, 8.51 G of raw reads (581,816 reads) were obtained, and 7.76 G clean reads were remained after quality control (530,616 reads). The average read length of filtered reads was 15.71 kb (N50 = 22.12 kb).

### Organelle genome assembly

For the plastome assembly, we utilized GetOrganelle (v1.7.4.1) to assemble the Illumina short-reads (Jin et al., 2020) with the following parameters of ‘-R 15 -k 21,45,65,85,105 -F embplant_pt’. Subsequently, we employed Flye (v.2.9.1-b1780) to perform *de novo* assembly of the long-reads from *A. albus* using the parameters of ‘–min-overlap 1,000’. For the assembled contigs, the draft mitogenome based on Nanopore long-reads was identified using BLASTn (Chen et al., 2015) program. Specifically, we used makeblastdb to construct a database for the assembled sequences by Flye, and then we used the conserved mitochondrial genes from *Liriodendron tulipifera* (NC_021152.1) as the query sequence to identify contigs containing conserved mitochondrial genes. All potential mitochondrial contigs were identified, and we subsequently mapped the short-reads to these contigs, retaining all mapped reads using BWA and SAMTools (Li and Durbin, 2009; Li et al., 2009). Considering the presence of homologous regions between the mitogenome and chloroplast sequence, it is highly probable that they were replaced by their chloroplast counterparts during polishing. Thus, we combined Illumina short-reads and Nanopore long-reads for hybrid assembly by using Unicycler (Wick et al., 2017), with the parameters of ‘–kmers 67, 77, 89, 105’. In this step, the mapped Illumina short-reads before were assembled by calling spades (Bankevich et al., 2012), and then, the Nanopore long-reads were used to resolve the regions of repetitive sequences of the assembly by calling minimap2 (Li, 2018). The resulting GFA format files generated by Unicycler were visualized using Bandage (Wick et al., 2015). We found that the mitogenome of *A. albus* contains multiple independent circular contigs. Here, we determine the complete assembly based on two principles. The first is that all core genes must be present, as the so-called ‘24 core genes’ are highly conserved with only a few exceptions reported. Secondly, all mitochondrial fragments need to be fully extended during assembly, particularly when dealing with linear contigs, we need to ensure that both ends of the contigs are fully extended until no new reads can be added to the extension. In our assembly, all conserved mitochondrial genes, including the 24 core genes, are annotated in the *A. albus* mitogenome. Computationally, all mitochondrial fragments are fully extended, either forming circular structures or closed structures with a net-like branching, indicating that our assembly contains all mitochondrial sequences. In addition, we performed a BLAST search on all circular contigs assembled that did not have any mitochondrial genes annotated in order to identify potential mitochondrial sequences. Specifically, for circular contig 13, it had no mitochondrial gene annotations, but due to its sequencing depth being close to that of other mitochondrial sequences and having the best hit with mitochondrial sequences on NCBI database, it was retained. We believe that this strategy will further help us obtain the complete mitochondrial genome sequence of such a multichromosomal species. Ultimately, we obtained a total of nineteen circular contigs (chromosomes) representing the complete mitogenome of *A. albus.*

### Mitogenome annotation

We firstly utilized the custom program IPMGA (http://www.1kmpg.cn/mgavas) to annotate the mitogenome. And then GeSeq (Tillich et al., 2017) was used to annotate the mitogenome of *A*. *albus* with two reference mitogenomes from GenBank: *Liriodendron tulipifera* (NC_021152.1) and *Spirodela polyrhiza* (NC_017840.1). The tRNA and rRNA annotations were performed using tRNAscan-SE (Lowe and Eddy, 1997) and BLASTn (Chen et al., 2015), respectively. Subsequently, we manually edited the annotations with any identified issues using Apollo (Lewis et al., 2002) and finally generated the genome map using OGDRAW (version 1.3.1).

### Repetitive elements

The simple sequence repeats (SSRs) in the assembled mitogenome were identified using the online website MISA (https://webblast.ipk-gatersleben.de/misa/). The minimum numbers of mono-, di-, tri-, tetra-, penta-, and hexanucleotides were set as 10, 5, 4, 3, 3, and 3, respectively. Furthermore, forward, reverse, palindromic, and complementary repeat sequences were identified using REPuter (Benson, 1999) (https://bibiserv.cebitec.uni-bielefeld.de/reputer/) with the following settings: a hamming distance of three and minimal repeat size of 30 bp. The e-value was limited to less than 1*e*-05. To further identify the dispersed repetitive sequences located on different chromosomes, we connected the chromosomes for analysis, and then allocated the results according to the position of each chromosome in the concatenated sequence. The visualization of the repetitive elements was performed using the Circos package (Zhang et al., 2013).

### Identification of the mitochondrial plastid sequences

To identify the mitochondrial plastid sequences (MTPTs), we compare the plastome and mitogenome sequences of *A. albus* by using BLASTn (Chen et al., 2015) program with the following parameters: -evalue 1*e*-5, -word_size 9, -gapopen 5, - gapextend 2, -reward 2, -penalty -3. The BLASTn (Chen et al., 2015) results were visualized using Circos package (Zhang et al., 2013). The identified MTPTs were also annotated by using GeSeq. We did not include duplicate counting in our analysis, such as the two MTPTs located on the inverted repeat regions of the plastome.

### Prediction of RNA editing sites

The Deepred-mt (Edera et al., 2021) was used for the prediction of C to U RNA editing sites of the mitochondrial PCGs. This tool makes predictions based on the convolutional neural network (CNN) model, and it has a high accuracy compared with previous prediction tools. We chose results with probability values above 0.9.

### Phylogenetic analysis

We downloaded the mitogenomes of 29 closely related species and one outgroup (*Amborella trichopoda*) from NCBI (https://www.ncbi.nlm.nih.gov/) nucleotide database based on the kinship of the *A. albus*. A total of 31 orthologous PCGs among the analyzed species were identified and extracted by using PhyloSuite (v.1.2.2) (Zhang et al., 2020). The corresponding nucleotide sequences were aligned using MAFFT (v7.471) (Katoh and Standley, 2013). Subsequently, the aligned sequences were concatenated and used to construct the phylogenetic tree. The maximum likelihood (ML) method was implemented in RAxML (v.8.2.4). The parameters were “raxmlHPC-PTHREADS-SSE3 -f a -N 1000 -m GTRGAMMA -x 551314260 -p 551314260”. The bootstrap analysis was performed with 1,000 replicates. The phylogenetic tree was edited on the online website ITOL (Letunic and Bork, 2019).

### Colinear analysis

We selected six additional species *Triticum aestivum* (NC_036024.1), *Phoenix dactylifera* (NC_016740.1), *Spirodela polyrhiza* (NC_017840.1), *Butomus umbellatus* (NC_021399.1), *Stratiotes aloides* (NC_035317.1) and *Liriodendron tulipifera* (NC_021152.1) to conduct a colinear analysis with *A. albu.* Colinear blocks were identified based on sequence similarity using BLASTn (Chen et al., 2015) program, employing the following parameters: -evalue 1*e*-5, -word_size 9, -gapopen 5, - gapextend 2, -reward 2, -penalty -3. Only colinear blocks longer than 500 bp were considered for subsequent analysis. The multiple synteny plot was drawn using TBtools (Chen et al., 2020) by calling the MCscan (Wang et al., 2012) source program.

### Development and validation of molecular markers for *A. albus*

Firstly, we downloaded whole genome sequencing (WGS) data for five *Amorphophallus* species from the SRA database, including: SRR7938681 (*A. konjac*), SRR7938682 *(A. muelleri*), SRR7938683 (*A. albus*), SRR5626705 (*A. paeoniifolius*), and SRR7938684 (*A. bulbifer*). The intron regions of all mitochondrial PCGs of *A. albus* were used as reference sequences to filtering WGS reads. Subsequently, all mapped reads were assembled to generate corresponding intron regions using spades (Bankevich et al., 2012). The alignment of these intron regions was performed using MAFFT (v7.450) (Rozewicki et al., 2019). The alignments revealed conservation among most intron regions (data not shown). Only two potentially highly variable intron regions, *nad2i156* and *nad4i976* were selected for subsequent validation.

The primers were designed using the Primer designing tool on NCBI (https://www.ncbi.nlm.nih.gov/tools/primer-blast/), the parameters are default. We sampled five *Amorphophallus* species (*A. albus, A. konjac, A. krausei, A. bulbifer*, and *A. paeoniifolius*), and collected three individuals with different source of habitats as three replicates for each of them. Then we extracted DNA and the amplifications were carried out in a Pro-Flex PCR system (Applied Biosystems, Waltham, MA, USA). The final volume of PCR amplification is 25 µL, including 2 µL template DNA, 1 µL forward primer, 12.5 µL 2×Taq PCR Master Mix and 9.5 µL ddH2O. The following amplifications conditions: denaturation at 94°C for 5 min. 30 cycles for 30 s at 94°C, 30 s at 58°C, 60 s at 72°C and 72°Cfor 5 min as the final extension. The PCR amplicons were visualized by 1% agarose gels electrophoresis, and the distinct bright bands were excised and sent to the Sangon Biotech (Shanghai) Co., Ltd for Sanger sequencing.

## Results

### Characteristic of the *A*. *albus* mitogenome

We employed Unicycler to assemble the mitogenome of *A*. *albus*, and the graphical representation of the assembly was visualized using Bandage software (**Figure S1A**). The assembly comprised 46 nodes, with each node representing an assembled contig. The nodes exhibited overlapping regions along connected lines. Notably, based on depth coverage analysis, we identified several predicted duplicated sequences, namely nodes 4, 8, 7, 13, 16, 21, 26, 29, 32, and 37. Additionally, node 1, node 11, node 18, node 24, node 31, and 40 were predicted to be sequences migrated from the chloroplast genome, as they displayed high sequence similarity to the mitogenome and were indistinguishable during the assembly process. The length and coverage of depth for each node are shown in **Table S1**. By mapping the Nanopore long-reads to the repetitive nodes, the graphic assembly is simplified to 19 circular contigs (**Figure S1B**), representing the complete mitogenomes of *A*. *albus*. Here, we called the simplified contigs as chromosomes. The detailed paths of each chromosome can been found in **Table S2**. The total length of *A*. *albus* mitogenome is 537,044 bp, with a GC content of 46.19%. The length of the longest chromosome is 56,458 bp, while the shortest measures 12,040 bp. For additional details on the accession number, length and GC content of each chromosome, please refer to **Table 1**. The accuracy of the mitogenome assembly was confirmed by mapping the Illumina short-reads onto the assembly, with a ~30-fold mean average of depth (**Figure S2**). As shown in **Figure S2**, every base on the 19 chromosomes was covered by short reads, which indicates that our assembly results were correct.

**Table 1:** The information of plastome and mitogenome of *A. albus*.

mitogenome	Length (bp)	GC content (%)	Accession Number
Circular 1	56,458	45.00	OM066869
Circular 2	48,380	45.43	OM066870
Circular 3	40,131	47.01	OM066871
Circular 4	39,352	46.63	OM066872
Circular 5	35,805	46.33	OM066873
Circular 6	34,782	46.41	OM066874
Circular 7	32,048	46.86	OM066875
Circular 8	29,468	45.53	OM066876
Circular 9	28,742	47.04	OM066877
Circular 10	27,358	46.88	OM066878
Circular 11	26,831	47.19	OM066879
Circular 12	24,194	46.30	OM066880
Circular 13	20,670	45.12	OM066881
Circular 14	19,531	46.62	OM066882
Circular 15	16,969	47.00	OM066883
Circular 16	16,584	46.13	OM066884
Circular 17	15,495	46.81	OM066885
Circular 18	12,224	44.79	OM066886
Circular 19	12,040	43.88	OM066887
Plastome	175,728	34.94	OP531918

### Mitogenome annotation

**Figure 1:**
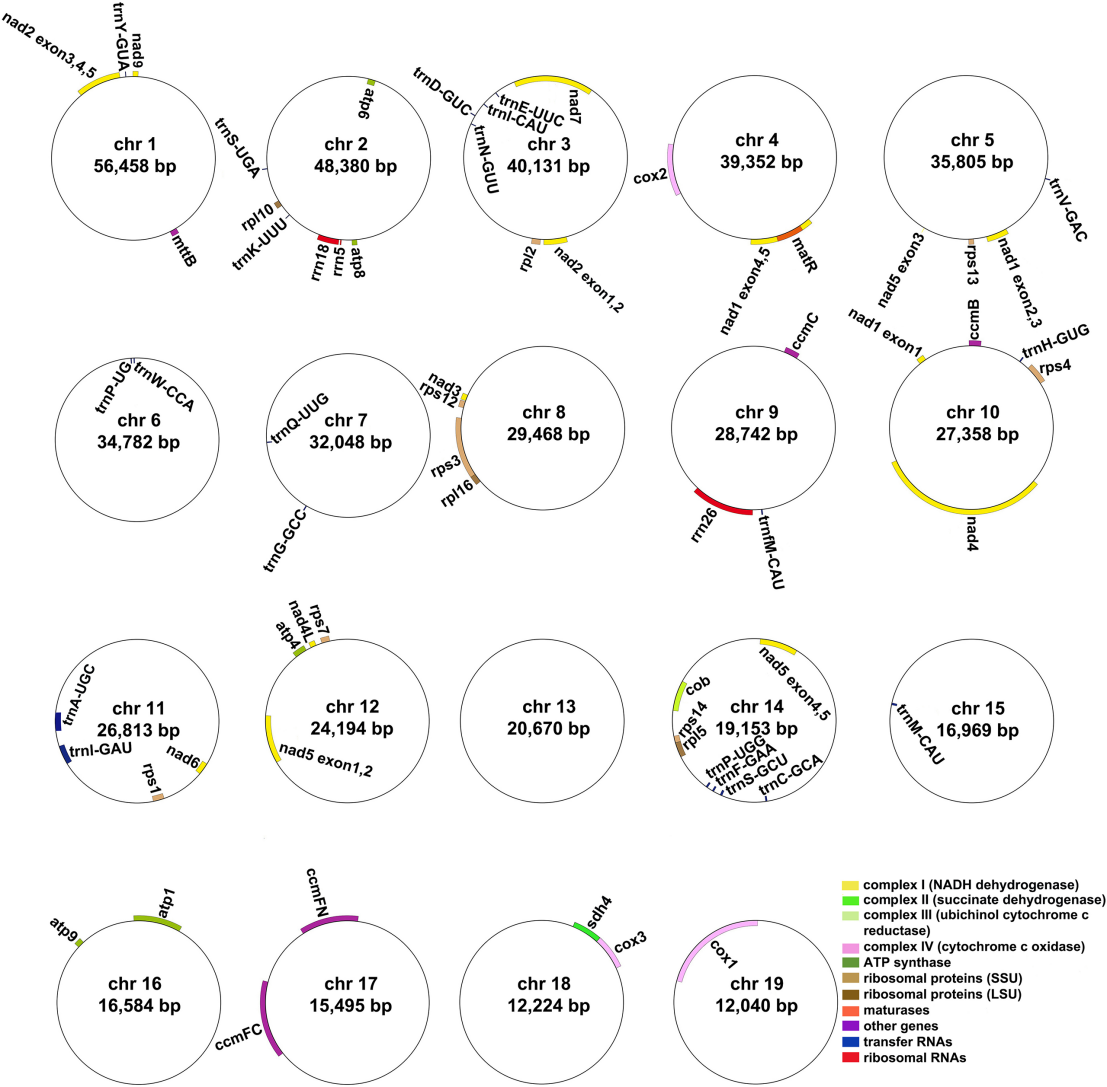
The mitogenome maps of *A. albus*. The mitogenome consists of nineteen circular chromosomes with different lengths and gene contents. Genes transcript clockwise or counter-clockwise strands are drawn on the upper or lower of the circles, respectively. Genes belonging to different functional groups are color-coded.

The *A*. *albus* mitogenome contains a total of 36 unique protein-coding genes (PCGs), which consist of 24 unique core genes and 12 unique variable genes. The 24 core genes include five ATP synthase genes (*atp1*, *atp4*, *atp6*, *atp8*, and *atp9*), one cytochrome *b* gene (*cob*), nine NADH dehydrogenase genes (*nad1*, *nad2*, *nad3*, *nad4*, *nad4L*, *nad5*, *nad6*, *nad7*, and *nad9*), three cytochrome *c* oxidase subunits (*cox1*, *cox2*, and *cox3*), one transport membrane protein gene (*mttB*), one maturases gene (*matR*), and four cytochrome *c* biogenesis genes (*ccmB, ccmC, ccmFC, and ccmFN*). The 12 unique variable genes consist of three large subunits of ribosome genes (*rpl5*, *rpl10*, and *rpl16*), eight small subunits of ribosome genes (*rps1*, *rps2*, *rps3*, *rps4*, *rps7*, *rps12*, *rps13*, *rps14*) and one succinate dehydrogenase gene (*sdh4*). Additionally, we annotated 21 tRNA genes (21 unique tRNA genes). The *trnV-GAC, trnP-UGG, trnW-CCA, trnH-GUG, trnA-UGC, trnI-GAU*, and *trnM-CAU* are plastid-derived, the other tRNA genes are mitochondria-native. Furthermore, three unique rRNA genes were identified: *rrn5*, *rrn18*, and *rrn26*. The detailed location of each gene is given in **Table S3**. **Figure 1** illustrates the genome maps of nineteen chromosomes. Among the annotated genes of *A*. *albus*, 11 genes contain introns (**Table S3**). Specifically, gene *ccmFC*, *cox1*, *rps3*, *trnA-UGC*, and *trnI-GAU* have one intron, *cox2* has two introns, *nad4* has three introns, and *nad1*, *nad2*, *nad5*, and *nad7* have four introns.

### Repeat elements

**Figure 2:**
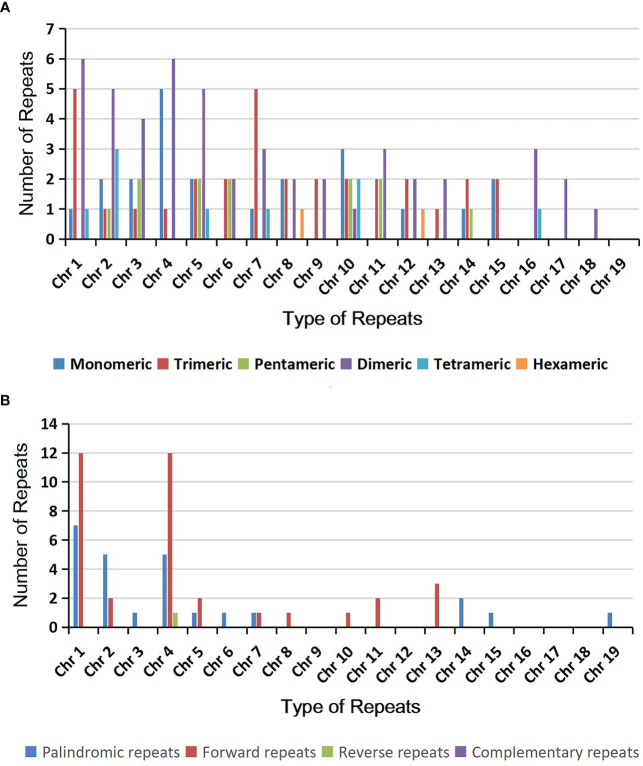
The simple sequence repeats (SSRs) and dispersed repeats identified in the mitogenomes of *A. albus.* **(A)** The identified SSRs on the mitogenome of *A. albus*. Each column represents different nucleotide repeat units displayed in different colors. The numbers of repetitive sequences in each category are shown on the top of the corresponding columns. **(B)** Dispersed repeats (≥ 30bp, distributed within the same chromosome) identified on the nineteen chromosomes.

Microsatellites, also known as simple repeat sequences or SSRs, are commonly found in eukaryotic genomes and typically consist of tandem repeats of 6 base pairs. In the *A*. *albus* mitogenome, we identified a total of 126 SSRs across the nineteen chromosomes (**Table S4**), with tetrameric repeats being the most abundant. Monomeric and dimeric SSRs accounted for 42.86% of the total SSRs. Monomeric thymine (T) repeats accounted for 63.64% (14) of the 22 monomeric SSRs, CT repeats accounted for 21.88% (7) of the 32 dimeric SSRs. And there are also 12 trimeric SSRs, 49 tetrameric SSRs, 9 pentameric SSRs and 2 hexametric SSRs. The SSRs distribution on each circular chromosome is shown in **Figure 2A**.

**Figure 3:**
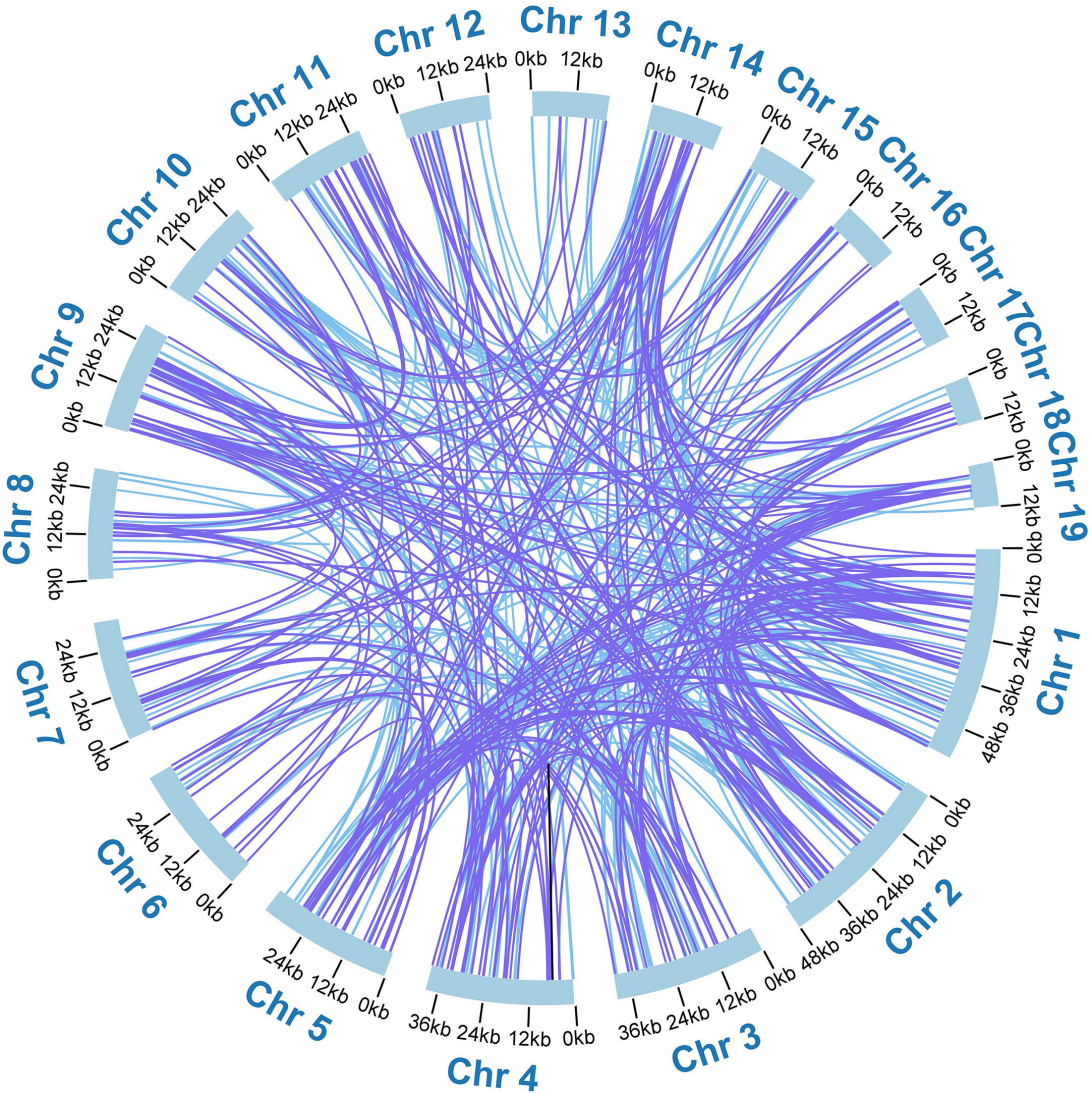
The distribution of dispersed repeats among the nineteen chromosomes. Arcs connect similar repeats within and between chromosomes; Purple arcs represent 250 forward repeats, blue represents 267 palindromic repeats and black represents 1 reverse repeat.

Additionally, we detected 518 pairs of dispersed repeats with lengths greater than or equal to 30 across the nineteen chromosomes, including 250 pairs of forward repeats, 267 pairs of palindromic repeats, and 1 pair of reverse repeats (**Table S5**). The number of dispersed repeats was significantly higher than that of SSRs. While most of these repeats were less than 100 bp in length, the *A. albus* mitogenome contained seventeen repeats longer than 100 bp, with the longest repeat spanning 3,445 bp. Interestingly, the dispersed repeats were not only found within the same chromosome but also distributed among different chromosomes. The distribution of dispersed repeats within the same chromosome is illustrated in **Figure 2B**. Collectively, the total length of these dispersed repeats is 28,048 bp, accounting for 5.23% of the whole mitogenome of *A*. *albus*, which showed an abundance of repeats. We utilized the Circos package (Krzywinski et al., 2009) to visually represent the distribution of dispersed repeats across nineteen chromosomes (**Figure 3**). These repeats are likely to play a role in genome rearrangement and may contribute to genome size variation.

### Analysis of mitochondrial plastid DNA sequences

**Figure 4:**
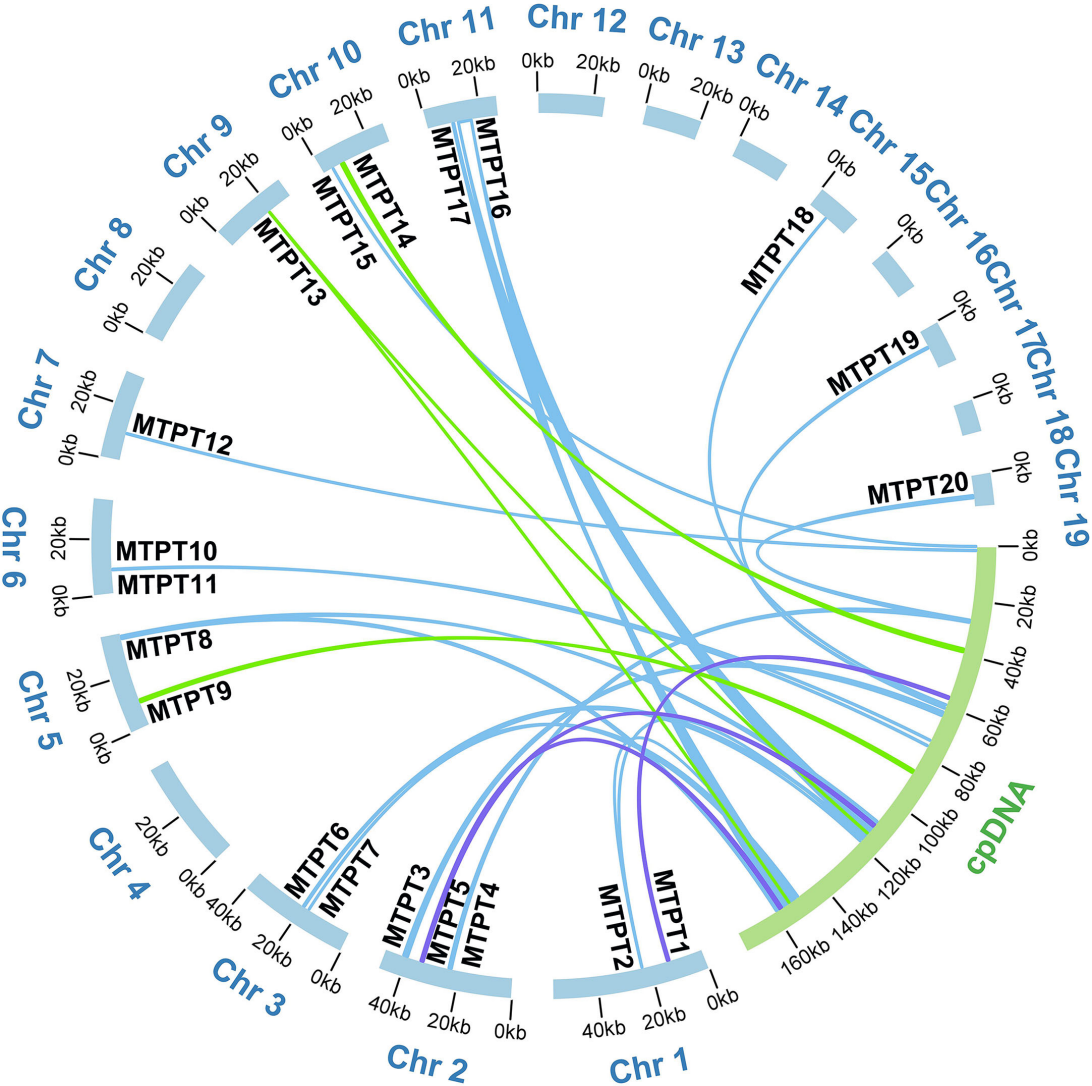
Schematic representation of the distribution of MTPTs between the nineteen mitogenome chromosomes and the plastome of *A. albus*. The MTPTs on the chloroplast IR regions were counted only once. Different colors of ribbons represent different identities: purple: 70%-80%, green: 80%-90%, and blue: 90%-100%.

The mitogenome of higher plants has extensive sequence migrated from its plastome and even from nuclear genomes. In our study, we annotated the chloroplast genome of *A*. *albus* and compared it with its mitogenome. By using BLASTn (Chen et al., 2015) program, we identified a total of 20 MTPTs between the two organelle genomes. These 20 MTPTs have a combined length of 22,421 bp, accounting for 4.17% of the mitogenome (**Table S6**) and 12.76% of the plastome. Among these MTPTs, MTPT3, MTPT8, MTPT14, MTPT16 exceeded 1,000 bp in length. MTPT16 is the longest, spanning 4,808 bp, while MTPT2 is the shortest with only 38 bp. We further annotated these MTPTs and found that all of them contained plastidial genes. As shown in **Table S6**, MTPT16 harbored two complete tRNA genes (*trnA-UGC, trnI-GAU*) and 2 partial rRNA genes (*rrn16S*, *rrn23S*). MTPT8, MTPT10, MTPT11, MTPT15, MTPT16, and MTPT18 contained seven complete tRNA genes, namely *trnV-GAC*, *trnP-UGG*, *trnW-CCA*, *trnH-GUG*, *trnI-GAU, trnA-UGC* and *trnM-CAU*. Besides, MTPT1, MTPT3, MTPT4, MTPT9, MTPT12, MTPT14, and MTPT19 contained incomplete plastidial PCGs, namely *nahC, ndhK*, *rpoB*, *rpoA, rps11*, *psbA*, *psbC*, *psbD*, and *atpB*. MTPT3 contained a partial plastidial gene (*rbcL*). The remaining MTPTs contained plastid ribosome RNA genes. **Figure 4** provides a schematic representation of the MTPTs. The results indicate that the above seven tRNA genes have migrated from the plastid genome to the mitogenome with minimal nucleotide substitutions. Conversely, the transferred PCGs have experienced varying degrees of sequence loss, and only partial sequences could be detected. This suggests that these PCGs may be non-functional.

### Prediction of RNA editing sites

**Figure 5:**
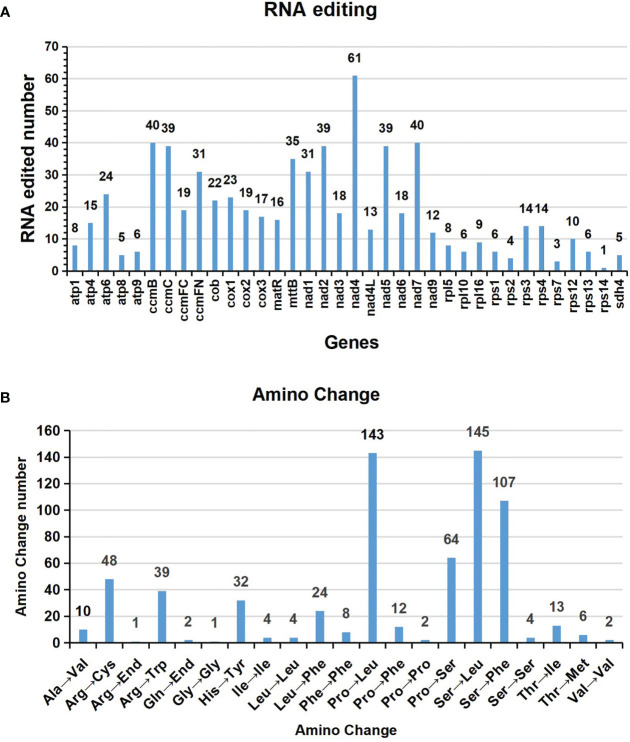
Characteristics of the RNA editing sites identified in mitochondrial PCGs of *A. albus.* The ordinate shows the number of RNA editing sites identified in PCGs, the abscissa shows the name of PCGs identified in the mitogenome of *A. albus*.

Based on Deepred-mt, we identified a total of 676 potential C to U RNA editing sites in 36 mitochondrial PCGs (**Table S7**). The predicted RNA editing sites for each gene are shown in **Figure 5A**. Among these mitochondrial genes, we identified 61 RNA editing sites in *nad4* and 40 for *ccmB* and *nad7* genes, which were the most top three among these PCGs. Additionally, *ccmC, nad2, nad5, mttB, ccmFN*, and *nad1* had more than 30 editing sites each. In contrast, the gene *rps14* had only one C to U editing sites, the fewest among them.

The interesting thing is that the stop codons of *atp6* and *ccmFC* are created by RNA editing, *atp6* is CAA to UAA, *ccmFC* is CGA to UGA. The start codons of *nad1* and *nad4L* are created by RNA editing, which was achieved by editing ACG to AUG.

**Figure 5B** shows amino acid changes resulting from RNA editing events. A total of 651 editing events resulted in amino acid changes, which means that 96.3% of edits result in non-synonymous substitutions. The most RNA editing events cause serine (Ser) and proline (Pro) being replaced with leucine (Leu) during translation, which occurred 146 and 145 times, respectively. Moreover, serine (Ser) was replaced by phenylalanine (Phe) 107 times, and the above three accounting for 61.14% of the total.

### Colinear analysis

**Figure 6:**
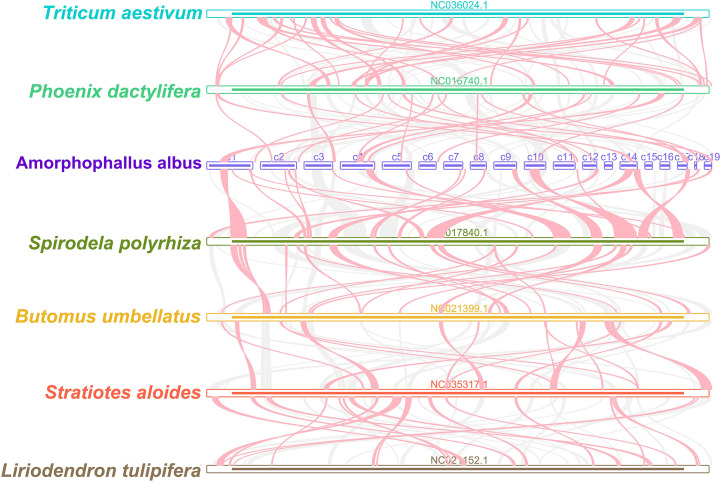
Mitogenome synteny. Bars indicated the mitogenomes, and the ribbons showed the homologous sequences between the adjacent species. The gray ribbons indicate regions with homology and the red ribbons indicate where the inversion occurred. The homologous blocks less than 0.5 kb in length are not remained, and regions that fail to have a homologous block indicate that they are unique to the species.

We identified the homologous regions between *A*. *albus* and its closely related species. The ribbon connecting the two genomes represents highly homologous collinear block (sequences). However, as shown in **Figure 6**, the mitogenomes exhibit poor collinearity, with numerous regions lacking homology between these mitogenomes. Furthermore, the colinear blocks are rearranged in a different order. These results suggest extensive genomic rearrangement between *A*. *albus* and the related mitogenomes, indicating that the genomic structure of the mitogenomes is not conserved.

### Phylogenetic analysis

**Figure 7:**
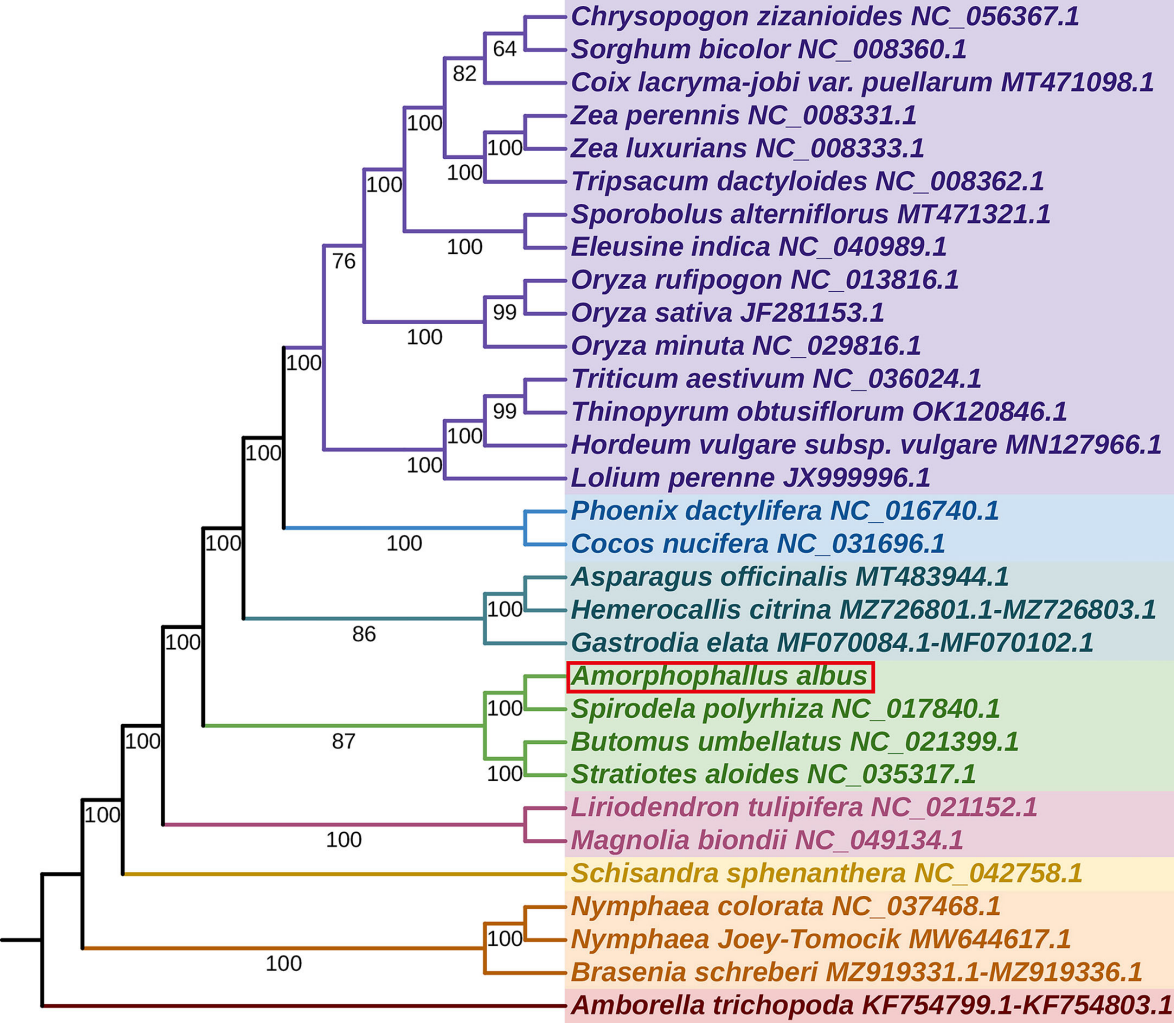
The phylogenetic relationships of *A. albus.* The tree was constructed based on the nucleotide sequences of 35 conserved mitochondrial protein-coding genes (PCGs), including *atp1, atp4, atp6, atp8, atp9, ccmB, ccmC, ccmFC, ccmFN, cob, cox1, cox2, cox3, matR, mttB, nad1, nad2, nad3, nad4, nad4L, nad5, nad6, nad7, nad9, rpl5, rpl10, rpl16, rps1, rps2, rps3, rps4, rps7, rps12, rps13*, and *rps14*. We used Maximum Likelihood (ML) method to reconstruct the phylogenetic tree. The ML topology is indicated with ML bootstrap support values. *Amborella trichopoda* was used as an outgroup.

We constructed a phylogenetic tree using 31 angiosperm species’ mitogenomes, with *Amborella trichopoda* serving as the outgroup. The species list and GenBank accessions used for phylogenetic analysis are shown in **Table S8**. For the analysis, we used the 24 core PCGs as the common genes, and the concatenated 24 aligned nucleotide sequences were used as the matrix data. Phylogenetic analyses yielded a well-supported phylogenetic tree, with most of the nodes having Maximum Likelihood (ML) bootstrap support values > 90, showing the reliability of the recovered phylogeny (**Figure 7**). The result revealed that *A*. *albus* was closely related to *Spirodela polyrhiza*, the two species belonging to the family Araceae. Overall, the topology is similar to that of APG IV system.

### Molecular marker development based on *A. albus* mitogenome

**Figure 8:**
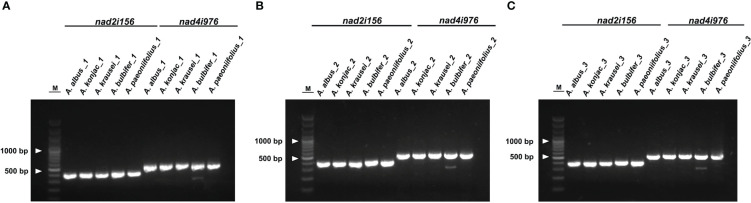
The gel electrophoresis results of DNA barcodes using designed primers. M, 3000-bp marker. **(A)** represent the result of the first replicate, **(B)** represent the result of the second replicate, and **(C)** represent the result of the third replicate. The bands in each experiment from left to right corresponded to products amplificated of *A. albus*, *A. konjac*, *A. krausei*, *A. bulbifer* and *A. paeoniifolius*.

**Figure 9:**
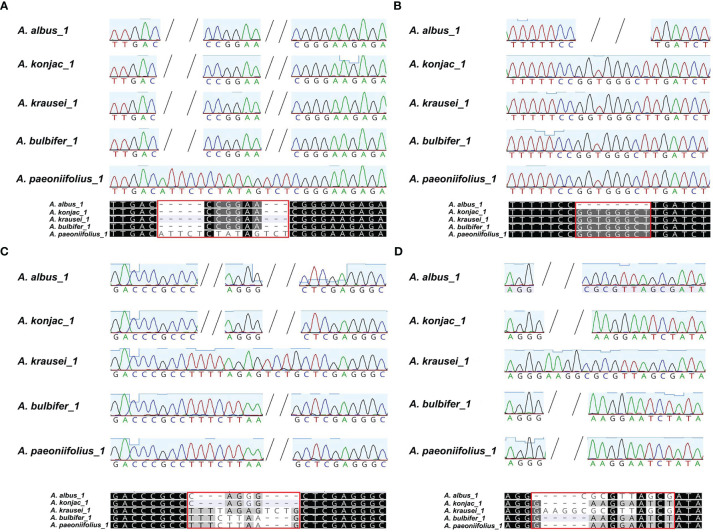
The alignment of the sanger reads of the PCR products. The nucleotides identical across all alignment are shaded in black, whereas those conserved in 60 % of the sequences are shaded in gray. The short horizontal lines indicate Indels. Each red box marks the variable locus. **(A, B)** are the two variable loci extracted from the alignment of *nad2i156*, **(C, D)** are the two variable loci extracted from alignment of *nad4i976*. These four variable loci can distinguish all five species of Konjac.

To discriminate the five *Amorphophallus* species, we selected two hypervariable intron regions, that is *nad2i156* and *nad4i976*. The two pairs of designed PCR primers are shown in **Table S9**. As expected, the PCR amplification products’ sizes (**Figure 8**) and the Sanger sequencing results (**Figure S4**) from three independent replicate experiments were consistent. The reads obtained from Sanger sequencing of the three individuals for each species are identical in sequence. **Figure 9** presents the Sanger sequencing result from the first experiment, the sequencing results of the other two replicate experiments are the same, which is not shown here. The untreated electropherogram of the three replicate experiments can be found in **Figure S3**, while the complete alignment of Sanger sequencing is shown in **Figures S4**, **9A, B** present the alignment results for *nad2i156*, where **Figure 9A** reveals a 14 bp variable locus, and **Figure 9B** reveals an 8 bp variable locus. These two variable loci can differentiate *A. albus and A. paeoniifolius.* Meanwhile, **Figures 9C, D** showing the alignment of *nad4i976*, with a 12 bp variable locus and a 15 bp variable locus, respectively. These two variable loci can be used to differentiate three of the five *Amorphophallus* species, except *A. bulbifer* and *A. paeoniifolius.* However, combined these four variable loci, these five *Amorphophallus* species can be discriminated successfully.

## Discussion

Regarding the SSRs in the *A. albus* mitogenome, it is observed that all the monomeric SSRs consist A or T nucleotides, resulting in a higher A/T content compared to the G/C content. This higher A/T content aligns with the findings in many other plant species (Bi et al., 2020; Yang et al., 2021). The abundance of AT repeats in the *A. albus* mitogenomes may be attributed to this higher A/T content. This result holds potential as reference information for the development of molecular marker in *A*. *albus.* In the analysis of dispersed repeat sequences, most of the dispersed repeat sequences in the mitogenome of *A*. *albus* are between 30 and 39 bp (74.95%). Many plants have a significantly higher proportion of repeats in this length region (30-39 bp) than in other length regions. Plant mitogenomes are a complex and dynamic mixture of forms rather than a single circle. Homologous recombination mediated by repeats is a common occurrence in plant mitogenomes. Many large repeats may act as sites for inter- or intra-molecular recombination (Sloan, 2013; Gualberto et al., 2014), leading to the generation of multiple alternative arrangements or isoforms. Moreover, the frequency of recombination mediated by long repeats are often higher than that mediated by short repeats. The multiple chromosomes of *A. albus* mitogenome may also have emerged from the splitting of larger circular chromosomes. For example, in our assembly, some chromosomes can be merged by repeating sequences in the draft assembly (**Figure S1A**), although more long-reads support their fission in our case. Thus, the mitogenome recombination of *A. albus* is also an interesting topic in the future under the condition of high sequencing depth.

The horizontal gene transfer (HGT) among organellar genomes and its nuclear genome is common and plays an important role in plant evolution. In *A*. *albus*, we have discovered abundant sequences transferred from the chloroplast genome to mitogenome (**Table S6**; **Figure 4**). Among all MTPTs, MTPT16 is the longest. Large segments of MTPTs have been also reported previously. MTPTs with total length of 26.87 kb were found in *Suaeda glauca* (Cheng et al., 2021), accounting for 5.18% of its mitogenome. These large MTPTs are believed to have a broad impact on eukaryotic evolution and promote genetic diversity. Previous research has shown that tRNAs in plant mitochondria have different origins: one part of tRNAs is inherited from the ancestor of the mitochondria, and the other part is derived from chloroplasts by HGT (Sprinzl and Vassilenko, 2005). Based on sequence similarity, we can trace the tRNA genes transferred from plastid to mitochondria in *A. albus*. In *A. albus* mitogenome, *trnV-GAC* (cp), *trnP-UGG* (cp), *trnW-CCA* (cp), *trnH-GUG* (cp), *trnI-GAU* (cp)*, trnA-UGC* (cp), and *trnM-CAU* (cp) were potentially transferred from plastid, near one third of all tRNAs in mitogenome. MTPTs are frequently found in the mitogenomes of other angiosperms, and throughout evolutionary timescales, these transfer events have led to the acquisition of functional tRNAs, as evidenced by their widespread conservation across angiosperms (Oda et al., 1992; Chaw et al., 2008; Guo et al., 2016). Among these tRNA genes, *trnW-CCA* (cp) is commonly found in the mitogenome of other angiosperms, and these tRNA genes appear to be homologous to their chloroplast counterparts, except for *Amborella trichopoda*, which is currently the only angiosperm known to possess a *trnW-CCA* of mitochondrial origin (Rice et al., 2013). Additionally, *trnP-UUG* (cp), possibly also be functional, as reported by Richardson (Richardson et al., 2013). Previous studies have shown that *trnH-GUG* (cp) and *trnM-CAU* (cp) might still be functional in the plant mitogenome, and they were migrated into the mitogenome at an early stage (Joyce and Gray, 1989; Alverson et al., 2010). The last two tRNA, *trnA-UGC* (cp) and *trnI-GAU* (cp), may have recently migrated from the plastid genome along with a large fragment (MTPT16, with a length of 4,808 bp), and they also found in Poaceae species, such as *Thinopyrum obtusiflorum* and *Elymus sibiricus* (Xiong et al., 2021; Wu et al., 2022). Further evidence is needed to confirm whether they are still functional in mitogenome. Some fragments of DNA from chloroplasts usually carry some PCGs during their transfer to the mitogenome, which often becomes nonfunctional pseudogenes (Kitazaki et al., 2011), and this is consistent with what is observed in our case (**Table S6**). The plastome sequences are generally considered to be highly conserved. And the sequence dialogue between the plastomes and mitogenomes in plants usually has been regarded as a one-way road (Plastomes transfer to mitogenome). However, some studies have reported the transfer of mitochondrial sequences to the plastomes, such as *Asclepias syriaca* (Straub et al., 2013) and *Daucus carota* (Iorizzo et al., 2012). But in *A. albus*, we have not observed any evidence of mitochondrial sequence transfer to the plastome in *A. albus*.

RNA editing is widespread in the mitogenome of higher plants and is a crucial step for gene expression. It falls under the category of post-transcriptional modifications. In most angiosperms, the chemical nature of which is a deamination reaction in which a site-specific cytosine (C) is changed to uracil (U). By RNA editing, the homology of mitochondrial protein sequences between different species was improved. It can also generate start and stop codons that are not present in the genomic sequence, and the new start and stop codons are usually generated to encode proteins that are more conserved and homologous to the corresponding proteins of other species, thus allowing better expression of genes in mitochondria (Edera et al., 2018). RNA editing events can regulate gene expression in plant growth and development (Bock and Khan, 2004; Chen et al., 2011; Raman and Park, 2015). In our predictions, most of the RNA editing sites occurred at the first position or second position of the triplet codon, similar to the case in most plants (Grewe et al., 2014; Kovar et al., 2018; Bi et al., 2020; Li et al., 2021; Yang et al., 2021). The identification of RNA editing sites can also provide clues to predict the gene function of new codons. In *A. albus* mitogenome, the *rps14* gene only has one RNA editing site, which indicates that the *rps14* gene is extremely conserved.

*Amorphophallus* species have significant economic value and are widely cultivated. And there have been many studies on intraspecific or interspecific molecular markers of Konjac, and some results have been achieved (Zheng et al., 2013; Pan et al., 2015; Gholave et al., 2017; Yin et al., 2019). However, all these markers are based on nuclear or plastid DNA. Compared with nuclear DNA, organelle genomes have multiple copies and monophyletic inheritance, offering potential advantages in the development of molecular markers. However, one study noted that some plastid barcoding markers co-amplified the conserved MTPTs and caused a barcoding paradox, resulting in mis-authentication of botanical ingredients and/or taxonomic mis-positioning (Park et al., 2020). In *A. albus*, the 20 MTPTs in mitogenome has a total length of 22,421 bp, such many homologous sequences may lead to potential identification problems. In this study, we used two hypervariable regions of mitogenome to develop markers for identifying species. In this way we can effectively avoid barcoding paradox, ensure correct authentication and/or taxonomic positioning of plant. Molecular markers in the mitogenome can be used for comprehensive comparison of genetic material between populations and individuals, improving the accuracy and reliability of plant classification. The molecular markers designed in the intron region of mitogenome have been reported in genus *Acer* (Ma et al., 2022). Here, we found four variable loci derived from two intron region, namely *nad2i156* and *nad4i976*, and successfully distinguished the five *Amorphophallus* species (As shown in **Figures 9A, B**). The molecular markers developed in the mitogenome in this study can also be potentially applied to the identification of other *Amorphophallus* species.

## Conclusion

We successfully assembled the *A. albus* mitogenome, which contained of nineteen circular chromosomes. Through the analysis of its gene content, repeating elements, RNA editing sites and other basic characteristics, we have gained valuable insights into the *A albus* mitogenome. Additionally, we conducted phylogenetic inferences, further enhancing our understanding of this species’ mitogenome. Moreover, based on the intron region of mitochondrial genes, we successfully developed two pairs of molecular markers, which can be combined to achieve molecular identification of five cultivated Konjac species. These results provide a new idea for the molecular identification of Konjac species and shows the mitochondrial DNA has potential application value in plant species identification.

## Data availability statement

The datasets presented in this study can be found in online repositories. The names of the repository/repositories and accession number(s) can be found below: https://www.ncbi.nlm.nih.gov/genbank/, OP531918.1 https://www.ncbi.nlm.nih.gov/genbank/, OM066869-OM066887 https://www.ncbi.nlm.nih.gov/, PRJNA880734 https://www.ncbi.nlm.nih.gov/, SAMN30880967 https://www.ncbi.nlm.nih.gov/, SRR22894643.

## Ethics statement

We collected fresh leaf materials of five *Amorphophallus* species for this study. The study, including plant samples, complies with relevant institutional, national, and international guidelines and legislation. No specific permits were required for plant collection.

## Author contributions

JY conceived and designed the research. XZ conducted experiments. JL assembled and annotated the mitogenome. YS and JL analyzed the data. YS prepared figures and tables. YS carried out the comparative analysis. YS and JL wrote the manuscript. All authors contributed to the article and approved the submitted version.

## References

[B1] Alverson A. J. Wei X. Rice D. W. Stern D. B. Barry K. Palmer J. D. (2010). Insights into the evolution of mitochondrial genome size from complete sequences of citrullus lanatus and cucurbita pepo (Cucurbitaceae). Mol. Biol. Evol. 27 (6), 1436–1448. doi: 10.1093/molbev/msq029 20118192 PMC2877997

[B2] Bankevich A. Nurk S. Antipov D. Gurevich A. A. Dvorkin M. Kulikov A. S.. (2012). SPAdes: a new genome assembly algorithm and its applications to single-cell sequencing. J. Comput. Biol. 19 (5), 455–477. doi: 10.1089/cmb.2012.0021 22506599 PMC3342519

[B3] Benson G. (1999). Tandem repeats finder: a program to analyze DNA sequences. Nucleic Acids Res. 27 (2), 573–580. doi: 10.1093/nar/27.2.573 9862982 PMC148217

[B4] Bi C. Lu N. Xu Y. He C. Lu Z. (2020). Characterization and analysis of the mitochondrial genome of common bean (Phaseolus vulgaris) by comparative genomic approaches. Int. J. Mol. Sci. 21, (11). doi: 10.3390/ijms21113778 PMC7312688 32471098

[B5] Birky C. W. Jr. (1995). Uniparental inheritance of mitochondrial and chloroplast genes: mechanisms and evolution. Proc. Natl. Acad. Sci. U.S.A. 92 (25), 11331–11338. doi: 10.1073/pnas.92.25.11331 8524780 PMC40394

[B6] Bock R. Khan M. S. (2004). Taming plastids for a green future. Trends Biotechnol. 22 (6), 311–318. doi: 10.1016/j.tibtech.2004.03.005 15158061

[B7] Bolger A. M. Lohse M. Usadel B. (2014). Trimmomatic: a flexible trimmer for illumina sequence data. Bioinf. (Oxford England) 30 (15), 2114–2120. doi: 10.1093/bioinformatics/btu170 PMC4103590 24695404

[B8] Bonora M. De Marchi E. Patergnani S. Suski J. M. Celsi F. Bononi A.. (2014). Tumor necrosis factor-alpha impairs oligodendroglial differentiation through a mitochondria-dependent process. Cell Death Differ. 21 (8), 1198–1208. doi: 10.1038/cdd.2014.35 24658399 PMC4085526

[B9] Burger G. Gray M. W. Lang B. F. (2003). Mitochondrial genomes: anything goes. Trends Genet. 19 (12), 709–716. doi: 10.1016/j.tig.2003.10.012 14642752

[B10] Chaw S. M. Shih A. C. Wang D. Wu Y. W. Liu S. M. Chou T. Y. (2008). The mitochondrial genome of the gymnosperm cycas taitungensis contains a novel family of short interspersed elements, bpu sequences, and abundant RNA editing sites. Mol. Biol. Evol. 25 (3), 603–615. doi: 10.1093/molbev/msn009 18192697

[B11] Chen C. Chen H. Zhang Y. Thomas H. R. Frank M. H. He Y.. (2020). TBtools: an integrative toolkit developed for interactive analyses of big biological data. Mol. Plant 13 (8), 1194–1202. doi: 10.1016/j.molp.2020.06.009 32585190

[B12] Chen H. Deng L. Jiang Y. Lu P. Yu J. (2011). RNA Editing sites exist in protein-coding genes in the chloroplast genome of cycas taitungensis. J. Integr. Plant Biol. 53 (12), 961–970. doi: 10.1111/j.1744-7909.2011.01082.x 22044752

[B13] Chen Y. Ye W. Zhang Y. Xu Y. (2015). High speed BLASTN: an accelerated MegaBLAST search tool. Nucleic Acids Res. 43 (16), 7762–7768. doi: 10.1093/nar/gkv784 26250111 PMC4652774

[B14] Cheng Y. He X. Priyadarshani S. Wang Y. Ye L. Shi C.. (2021). Assembly and comparative analysis of the complete mitochondrial genome of suaeda glauca. BMC Genomics 22 (1), 167. doi: 10.1186/s12864-021-07490-9 33750312 PMC7941912

[B15] Devaraj R. D. Reddy C. K. Xu B. (2019). Health-promoting effects of konjac glucomannan and its practical applications: A critical review. Int. J. Biol. Macromol. 126, 273–281. doi: 10.1016/j.ijbiomac.2018.12.203 30586587

[B16] Edera A. A. Gandini C. L. Sanchez-Puerta M. V. (2018). Towards a comprehensive picture of c-to-U RNA editing sites in angiosperm mitochondria. Plant Mol. Biol. 97 (3), 215–231. doi: 10.1007/s11103-018-0734-9 29761268

[B17] Edera A. A. Small I. Milone D. H. Sanchez-Puerta M. V. (2021). Deepred-Mt: deep representation learning for predicting c-to-U RNA editing in plant mitochondria. Comput. Biol. Med. 136, 104682. doi: 10.1016/j.compbiomed.2021.104682 34343887

[B18] Fauron C. Casper M. Gao Y. Moore B. (1995). The maize mitochondrial genome: dynamic, yet functional. Trends Genet. 11 (6), 228–235. doi: 10.1016/s0168-9525(00)89056-3 7638905

[B19] Gholave A. R. Pawar K. D. Yadav S. R. Bapat V. A. Jadhav J. P. (2017). Reconstruction of molecular phylogeny of closely related amorphophallus species of India using plastid DNA marker and fingerprinting approaches. Physiol. Mol. Biol. Plants 23 (1), 155–167. doi: 10.1007/s12298-016-0400-0 28250592 PMC5313404

[B20] Grewe F. Edger P. P. Keren I. Sultan L. Pires J. C. Ostersetzer-Biran O.. (2014). Comparative analysis of 11 brassicales mitochondrial genomes and the mitochondrial transcriptome of brassica oleracea. Mitochondrion 19 Pt B, 135–143. doi: 10.1016/j.mito.2014.05.008 24907441

[B21] Gualberto J. M. Mileshina D. Wallet C. Niazi A. K. Weber-Lotfi F. Dietrich A. (2014). The plant mitochondrial genome: dynamics and maintenance. Biochimie 100, 107–120. doi: 10.1016/j.biochi.2013.09.016 24075874

[B22] Guo W. Grewe F. Fan W. Young G. J. Knoop V. Palmer J. D.. (2016). Ginkgo and Welwitschia mitogenomes reveal extreme contrasts in gymnosperm mitochondrial evolution. Mol. Biol. Evol. 33 (6), 1448–1460. doi: 10.1093/molbev/msw024 26831941

[B23] Iorizzo M. Grzebelus D. Senalik D. Szklarczyk M. Spooner D. Simon P. (2012). Against the traffic: the first evidence for mitochondrial DNA transfer into the plastid genome. Mob. Genet. Elements 2 (6), 261–266. doi: 10.4161/mge.23088 23481035 PMC3575419

[B24] Jackman S. D. Coombe L. Warren R. L. Kirk H. Trinh E. MacLeod T.. (2020). Complete mitochondrial genome of a gymnosperm, sitka spruce (Picea sitchensis), indicates a complex physical structure. Genome Biol. Evol. 12 (7), 1174–1179. doi: 10.1093/gbe/evaa108 32449750 PMC7486957

[B25] Jin J. J. Yu W. B. Yang J. B. Song Y. dePamphilis C. W. Yi T. S.. (2020). GetOrganelle: a fast and versatile toolkit for accurate *de novo* assembly of organelle genomes. Genome Biol. 21 (1), 241. doi: 10.1186/s13059-020-02154-5 32912315 PMC7488116

[B26] Joyce P. B. Gray M. W. (1989). Chloroplast-like transfer RNA genes expressed in wheat mitochondria. Nucleic Acids Res. 17 (14), 5461–5476. doi: 10.1093/nar/17.14.5461 2762145 PMC318170

[B27] Katoh K. Standley D. M. (2013). MAFFT multiple sequence alignment software version 7: improvements in performance and usability. Mol. Biol. Evol. 30 (4), 772–780. doi: 10.1093/molbev/mst010 23329690 PMC3603318

[B28] Kitazaki K. Kubo T. Kagami H. Matsumoto T. Fujita A. Matsuhira H.. (2011). A horizontally transferred tRNA(Cys) gene in the sugar beet mitochondrial genome: evidence that the gene is present in diverse angiosperms and its transcript is aminoacylated. Plant J. 68 (2), 262–272. doi: 10.1111/j.1365-313X.2011.04684.x 21699590

[B29] Kovar L. Nageswara-Rao M. Ortega-Rodriguez S. Dugas D. V. Straub S. Cronn R.. (2018). PacBio-based mitochondrial genome assembly of leucaena trichandra (Leguminosae) and an intrageneric assessment of mitochondrial RNA editing. Genome Biol. Evol. 10 (9), 2501–2517. doi: 10.1093/gbe/evy179 30137422 PMC6161758

[B30] Kozik A. Rowan B. A. Lavelle D. Berke L. Schranz M. E. Michelmore R. W.. (2019). The alternative reality of plant mitochondrial DNA: one ring does not rule them all. PloS Genet. 15 (8), e1008373. doi: 10.1371/journal.pgen.1008373 31469821 PMC6742443

[B31] Kroemer G. Reed J. C. (2000). Mitochondrial control of cell death. Nat. Med. 6 (5), 513–519. doi: 10.1038/74994 10802706

[B32] Krzywinski M. Schein J. Birol I. Connors J. Gascoyne R. Horsman D.. (2009). Circos: an information aesthetic for comparative genomics. Genome Res. 19 (9), 1639–1645. doi: 10.1101/gr.092759.109 19541911 PMC2752132

[B33] Letunic I. Bork P. (2019). Interactive tree of life (iTOL) v4: recent updates and new developments. Nucleic Acids Res. 47 (W1), W256–W259. doi: 10.1093/nar/gkz239 30931475 PMC6602468

[B34] Lewis S. E. Searle S. M. Harris N. Gibson M. Lyer V. Richter J.. (2002). Apollo: A sequence annotation editor. Genome Biol. 3 (12), RESEARCH0082. doi: 10.1186/gb-2002-3-12-research0082 12537571 PMC151184

[B35] Li H. (2018). Minimap2: pairwise alignment for nucleotide sequences. Bioinformatics 34 (18), 3094–3100. doi: 10.1093/bioinformatics/bty191 29750242 PMC6137996

[B36] Li H. Durbin R. (2009). Fast and accurate short read alignment with burrows-wheeler transform. Bioinformatics 25 (14), 1754–1760. doi: 10.1093/bioinformatics/btp324 19451168 PMC2705234

[B37] Li H. Handsaker B. Wysoker A. Fennell T. Ruan J. Homer N.. (2009). The sequence Alignment/Map format and SAMtools. Bioinf. (Oxford England) 25 (16), 2078–2079. doi: 10.1093/bioinformatics/btp352 PMC2723002 19505943

[B38] Li J. Xu Y. Shan Y. Pei X. Yong S. Liu C.. (2021). Assembly of the complete mitochondrial genome of an endemic plant, scutellaria tsinyunensis, revealed the existence of two conformations generated by a repeat-mediated recombination. Planta 254 (2), 36. doi: 10.1007/s00425-021-03684-3 34302538

[B39] Lowe T. M. Eddy S. R. (1997). tRNAscan-SE: a program for improved detection of transfer RNA genes in genomic sequence. Nucleic Acids Res. 25 (5), 955–964. doi: 10.1093/nar/25.5.955 9023104 PMC146525

[B40] Ma Q. Wang Y. Li S. Wen J. Zhu L. Yan K.. (2022). Assembly and comparative analysis of the first complete mitochondrial genome of acer truncatum bunge: a woody oil-tree species producing nervonic acid. BMC Plant Biol. 22 (1), 29. doi: 10.1186/s12870-021-03416-5 35026989 PMC8756732

[B41] Oda K. Kohchi T. Ohyama K. (1992). Mitochondrial DNA of marchantia polymorpha as a single circular form with no incorporation of foreign DNA. Biosci. Biotechnol. Biochem. 56 (1), 132–135. doi: 10.1271/bbb.56.132 1368126

[B42] Pan C. Gichira A. W. Chen J. M. (2015). Genetic variation in wild populations of the tuber crop amorphophallus konjac (Araceae) in central China as revealed by AFLP markers. Genet. Mol. Res. 14 (4), 18753–18763. doi: 10.4238/2015.December.28.24 26782525

[B43] Park H. S. Jayakodi M. Lee S. H. Jeon J. H. Lee H. O. Park J. Y.. (2020). Mitochondrial plastid DNA can cause DNA barcoding paradox in plants. Sci. Rep. 10 (1), 6112. doi: 10.1038/s41598-020-63233-y 32273595 PMC7145815

[B44] Raman G. Park S. (2015). Analysis of the complete chloroplast genome of a medicinal plant, dianthus superbus var. longicalyncinus, from a comparative genomics perspective. PloS One 10 (10), e0141329. doi: 10.1371/journal.pone.0141329 26513163 PMC4626046

[B45] Rice D. W. Alverson A. J. Richardson A. O. Young G. J. Sanchez-Puerta M. V. Munzinger J.. (2013). Horizontal transfer of entire genomes *via* mitochondrial fusion in the angiosperm amborella. Science 342 (6165), 1468–1473. doi: 10.1126/science.1246275 24357311

[B46] Richardson A. O. Rice D. W. Young G. J. Alverson A. J. Palmer J. D. (2013). The "fossilized" mitochondrial genome of liriodendron tulipifera: ancestral gene content and order, ancestral editing sites, and extraordinarily low mutation rate. BMC Biol. 11, 29. doi: 10.1186/1741-7007-11-29 23587068 PMC3646698

[B47] Roger A. J. Munoz-Gomez S. A. Kamikawa R. (2017). The origin and diversification of mitochondria. Curr. Biol. 27 (21), R1177–R1192. doi: 10.1016/j.cub.2017.09.015 29112874

[B48] Rozewicki J. Li S. Amada K. M. Standley D. M. Katoh K. (2019). MAFFT-DASH: integrated protein sequence and structural alignment. Nucleic Acids Res. 47 (W1), W5–w10. doi: 10.1093/nar/gkz342 31062021 PMC6602451

[B49] Skippington E. Barkman T. J. Rice D. W. Palmer J. D. (2015). Miniaturized mitogenome of the parasitic plant viscum scurruloideum is extremely divergent and dynamic and has lost all nad genes. Proc. Natl. Acad. Sci. U.S.A. 112 (27), E3515–E3524. doi: 10.1073/pnas.1504491112 26100885 PMC4500244

[B50] Sloan D. B. (2013). One ring to rule them all? genome sequencing provides new insights into the 'master circle' model of plant mitochondrial DNA structure. New Phytol. 200 (4), 978–985. doi: 10.1111/nph.12395 24712049

[B51] Sloan D. B. Alverson A. J. Chuckalovcak J. P. Wu M. McCauley D. E. Palmer J. D.. (2012). Rapid evolution of enormous, multichromosomal genomes in flowering plant mitochondria with exceptionally high mutation rates. PloS Biol. 10 (1), e1001241. doi: 10.1371/journal.pbio.1001241 22272183 PMC3260318

[B52] Sprinzl M. Vassilenko K. S. (2005). Compilation of tRNA sequences and sequences of tRNA genes. Nucleic Acids Res. 33 (Database issue), D139–D140. doi: 10.1093/nar/gki012 15608164 PMC539966

[B53] Straub S. C. Cronn R. C. Edwards C. Fishbein M. Liston A. (2013). Horizontal transfer of DNA from the mitochondrial to the plastid genome and its subsequent evolution in milkweeds (apocynaceae). Genome Biol. Evol. 5 (10), 1872–1885. doi: 10.1093/gbe/evt140 24029811 PMC3814198

[B54] Tang R. Liu E. Zhang Y. Schinnerl J. Sun W. Chen G.. (2020). Genetic diversity and population structure of *Amorphophallus albus*, a plant species with extremely small populations (PSESP) endemic to dry-hot valley of Jinsha River. BMC Genet. 21 (1), 102. doi: 10.1186/s12863-020-00910-x 32919456 PMC7488774

[B55] Tillich M. Lehwark P. Pellizzer T. Ulbricht-Jones E. S. Fischer A. Bock R.. (2017). GeSeq - versatile and accurate annotation of organelle genomes. Nucleic Acids Res. 45 (W1), W6–w11. doi: 10.1093/nar/gkx391 28486635 PMC5570176

[B56] van Loo G. Saelens X. van Gurp M. MacFarlane M. Martin S. J. Vandenabeele P. (2002). The role of mitochondrial factors in apoptosis: a Russian roulette with more than one bullet. Cell Death Differ. 9 (10), 1031–1042. doi: 10.1038/sj.cdd.4401088 12232790

[B57] Wallace D. C. Singh G. Lott M. T. Hodge J. A. Schurr T. G. Lezza A. M.. (1988). Mitochondrial DNA mutation associated with leber's hereditary optic neuropathy. Science 242 (4884), 1427–1430. doi: 10.1126/science.3201231 3201231

[B58] Wang S. Li D. Yao X. Song Q. Wang Z. Zhang Q.. (2019). Evolution and diversification of kiwifruit mitogenomes through extensive whole-genome rearrangement and mosaic loss of intergenic sequences in a highly variable region. Genome Biol. Evol. 11 (4), 1192–1206. doi: 10.1093/gbe/evz063 PMC6482417 30895302

[B59] Wang Y. Tang H. Debarry J. D. Tan X. Li J. Wang X.. (2012). MCScanX: a toolkit for detection and evolutionary analysis of gene synteny and collinearity. Nucleic Acids Res. 40 (7), e49. doi: 10.1093/nar/gkr1293 22217600 PMC3326336

[B60] Wick R. R. Judd L. M. Gorrie C. L. Holt K. E. (2017). Unicycler: resolving bacterial genome assemblies from short and long sequencing reads. PloS Comput. Biol. 13 (6), e1005595. doi: 10.1371/journal.pcbi.1005595 28594827 PMC5481147

[B61] Wick R. R. Schultz M. B. Zobel J. Holt K. E. (2015). Bandage: interactive visualization of *de novo* genome assemblies. Bioinformatics 31 (20), 3350–3352. doi: 10.1093/bioinformatics/btv383 26099265 PMC4595904

[B62] Wu X. Hu X. Chen X. Zhang J. Ren C. Song L.. (2022). Sequencing and characterization of the complete mitochondrial genome of thinopyrum obtusiflorum (DC.) banfi 2018 (Poaceae). Mitochondrial DNA B Resour. 7 (3), 539–540. doi: 10.1080/23802359.2022.2054378 35356795 PMC8959512

[B63] Xiong Y. Yu Q. Xiong Y. Zhao J. Lei X. Liu L.. (2021). The complete mitogenome of elymus sibiricus and insights into its evolutionary pattern based on simple repeat sequences of seed plant mitogenomes. Front. Plant Sci. 12. doi: 10.3389/fpls.2021.802321 PMC8826237 35154192

[B64] Yang H. Li W. Yu X. Zhang X. Zhang Z. Liu Y.. (2021). Insights into molecular structure, genome evolution and phylogenetic implication through mitochondrial genome sequence of gleditsia sinensis. Sci. Rep. 11 (1), 14850. doi: 10.1038/s41598-021-93480-6 34290263 PMC8295344

[B65] Ye N. Wang X. Li J. Bi C. Xu Y. Wu D.. (2017). Assembly and comparative analysis of complete mitochondrial genome sequence of an economic plant salix suchowensis. PeerJ 5, e3148. doi: 10.7717/peerj.3148 28367378 PMC5374973

[B66] Yin S. Yan Y. You L. Chen Q. Zhou Y. Chen K.. (2019). Newly developed genomic SSRs reveal genetic diversity in wild and cultivated *Amorphophallus albus* germplasms. Plant Mol. Biol. Rep. 37 (4), 365–375. doi: 10.1007/s11105-019-01162-5

[B67] Zhang D. Gao F. Jakovlic I. Zou H. Zhang J. Li W. X.. (2020). PhyloSuite: an integrated and scalable desktop platform for streamlined molecular sequence data management and evolutionary phylogenetics studies. Mol. Ecol. Resour. 20 (1), 348–355. doi: 10.1111/1755-0998.13096 31599058

[B68] Zhang H. Meltzer P. Davis S. (2013). RCircos: an r package for circos 2D track plots. BMC Bioinf. 14, 244. doi: 10.1186/1471-2105-14-244 PMC3765848 23937229

[B69] Zheng X. Pan C. Diao Y. You Y. Yang C. Hu Z. (2013). Development of microsatellite markers by transcriptome sequencing in two species of amorphophallus (Araceae). BMC Genomics 14, 490. doi: 10.1186/1471-2164-14-490 23870214 PMC3737116

