# Supplementary material for: The complete mitochondrial genome of Amorphophallus albus and development of molecular markers for five Amorphophallus species based on mitochondrial DNA
Source: Front Plant Sci. 2023 Jun 21;14:1180417. doi: 10.3389/fpls.2023.1180417 (PMC10322194; doi:10.3389/fpls.2023.1180417)

Figure S1. Schematic diagram of hybrid assembly based on Unicycler.

A. Graphical mitogenome assembly of *A. albus*. It contains 46 nodes, which are overlapping with each other along the connected lines. **B.** Nineteen circular chromosomes obtained by solving repetitive regions based on nanopore long-reads. The nineteen chromosomes represent the major conformations of the mitogenome. Each node represents an assembled unitig. The length and width of the nodes represent the relative length and average depth of coverage of the unitig, see Table S2 for detailed information of these nodes.

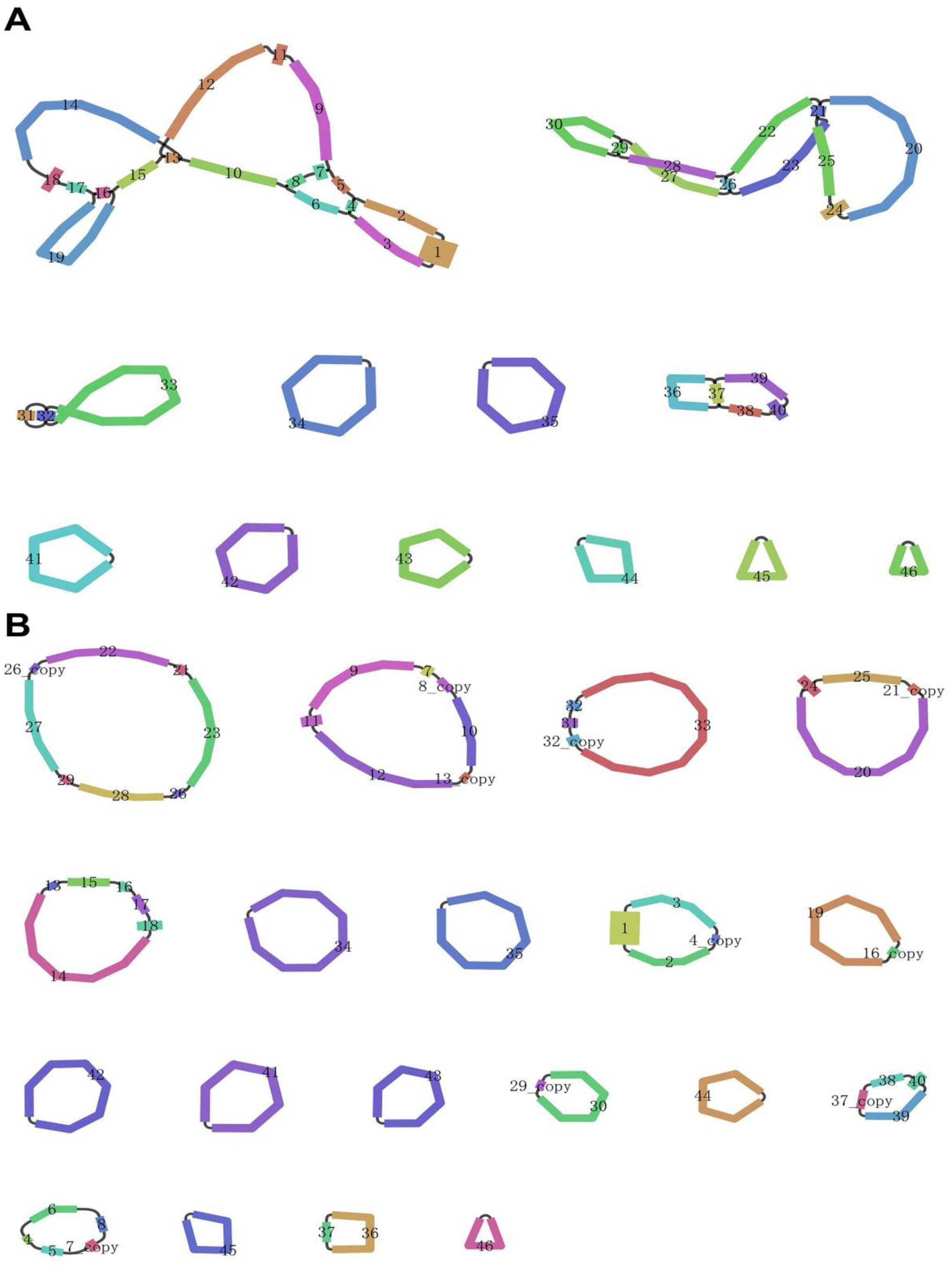

Figure S2. Sequencing depth of coverage based on Illumina short-reads.

A-S represent the depth of coverage from contig1 to contig19, respectively. The abscissa indicates the location of the contig, and the ordinate indicates the sequencing depth. The average depth of the nineteen contigs was about ~30×.

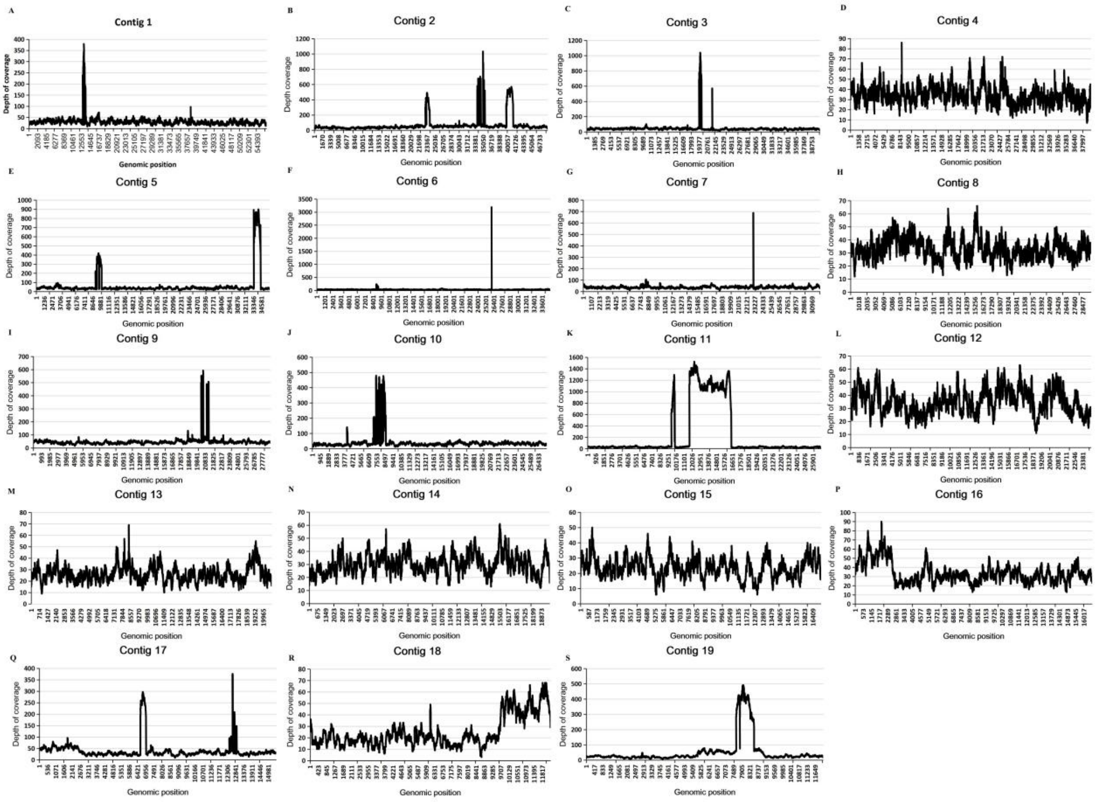

Figure S3. The original uncut electropherogram.

PCR-1 is the result of the first replicate, PCR-2 is the result of the second replicate, PCR-3 is the result of the third replicate.

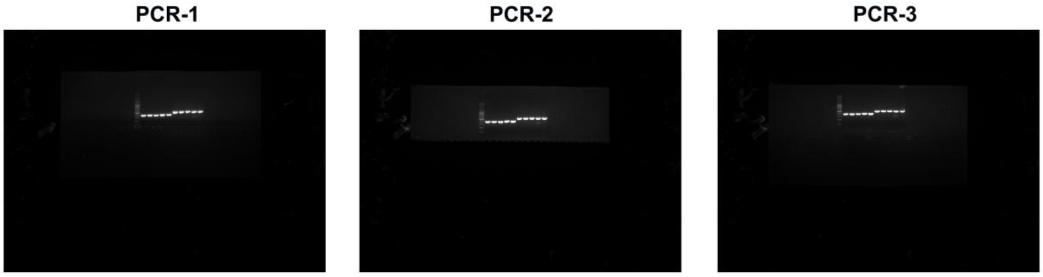

Figure S4. The full alignment of Sanger sequencing reads.

A. The alignment of parts of the amplified *nad2i156* regions, **B.** The alignment of parts of the amplified *nad4i976* regions. The red box shows partial variable loci that can be used to develop molecular markers to identify species.

A

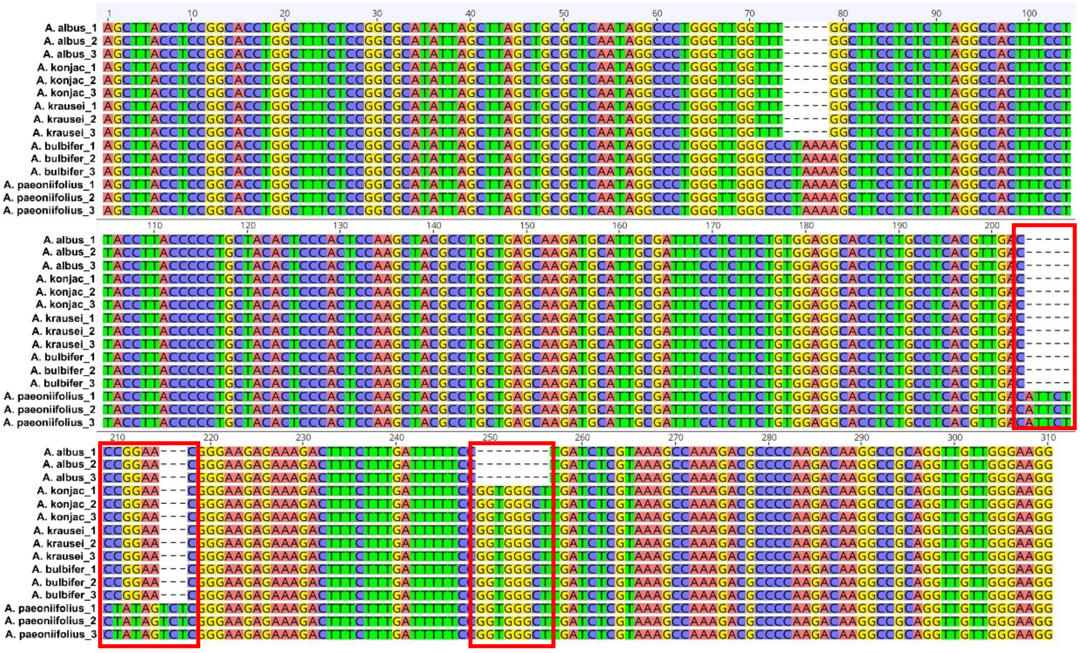

B

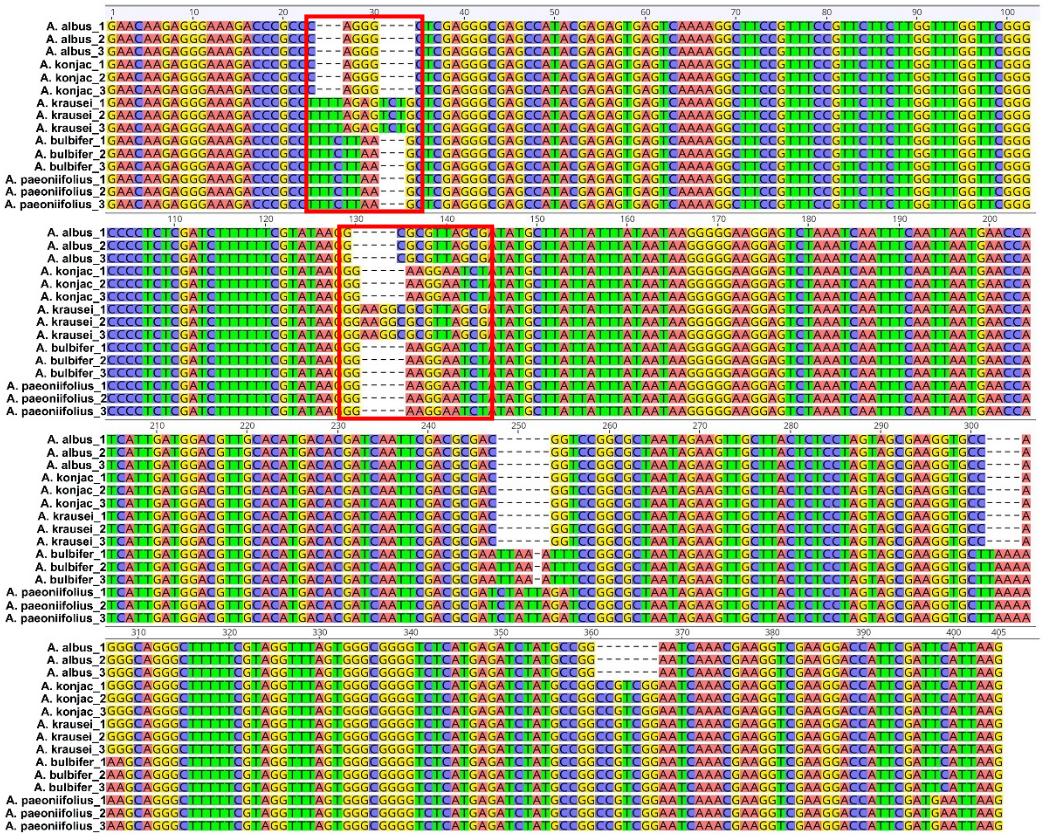

Supplement: Supplementary file 1 [file DataSheet_1.zip › Supplementary Figures 1 - 4.PDF]
